# Medicine

**Published:** 1896-03

**Authors:** Theodore Fisher


### Progress of the Medical Sciences.

### MEDICINE.

Physiologists have for some time differed as to whether the function of the supra-renal capsules is to render effete products of metabolism harmless, or to secrete some substance that is of value in the internal economy. The substance extracted from these bodies by Schäfer and Oliver, physiologically tested, caused great rise of arterial tension, and when the restraining action of the organ was abolished led to increase in force and rapidity of the heart-beat.

Since in **Addison's disease** low arterial tension and feeble cardiac action are such noteworthy features, it seemed reasonable to conclude that the destruction of the supra-renal capsules that is known to be present deprives the circulatory system of a necessary stimulant. Acting on this hypothesis, Dr. Oliver tried extract of supra-renal tissue in Addison's disease, and recorded two cases in which improvement took place. Other cases brought forward more recently seem to throw some doubt upon the efficacy of the remedy. Dr. Murrell [1] reports the case of a man aged 31, suffering from Addison's disease, in whom the symptoms appeared to be aggravated rather than lessened in severity by tabloids of supra-renal extract. Supra-renal glands, first of sheep then of calves, were substituted for the tabloids with no better result, and the patient steadily grew worse and died.. At the Clinical Society of London, Drs. Ringer and Phear[2] read notes of a case in which supra-renal extract also failed; and Dr. Ringer pointed out that should improvement occur in the course of the treatment, it does not follow that the extract has acted beneficially, since the course of Addison's disease may be variable and some temporary amelioriation take place. While these two cases are not encouraging, it must be mentioned that Dr. Sansom[3] records a case in which some improvement occurred under treatment with supra-renal extract, and a similar case is recorded by Dr. Lloyd Jones[4]. Stockton also[5] treated a case with benefit with the supra-renal glands of the sheep. The patient, a woman aged 40, gained weight, lost the pigmentation of the skin and her dyspeptic symptoms, while her pulse increased in force.

It would be disappointing were the treatment of Addison's disease by supra-renal extract to prove to be of no value, since the experiments of Oliver and Schäfer seemed to show that there was scientific ground for anticipating some beneficial result.

One is not surprised, however, when one hears of **bone-marrow** failing **in pernicious anæmia,** since it is not obvious how the marrow is expected to act. Bertram Hunt [6] gives notes of three cases of pernicious anæmia unsuccessfully treated with bone-marrow. During the time it was tried there was no increase of corpuscles or of hæmoglobin in the blood of any of the patients. Although bone-marrow failed, one of these cases is interesting as an illustration of the marked improvement that sometimes follows the use of arsenic. The patient was given

[1] *Lancet*, 1896, i. 289. [2] *Brit. M. J.*, 1896, i. 150.
[3] *Clin. Sketches*, 1895, ii. 132. [4] *Brit. M. J.*, 1895, ii. 483.
[5] *Med. News*, 1895, lxvii. 551. [6] *Lancet*, 1896, i. 282.

Fowler's solution, first in quantities of nine minims a day, rapidly rising to seventy minims. The hæmoglobin rose from 26 per cent. to 48 per cent. in three weeks. The large doses of arsenic caused the development of some peripheral neuritis, and the drug was discontinued. The improvement, however, was maintained, and the hæmoglobin reached 75 per cent. of the normal at the end of another fortnight, when the neuritis had apparently disappeared.

In connection with the treatment of disease by organic extracts, it may be mentioned that a few cases of recovery from **tetanus** continue to be reported under **antitoxin injections.** Delbecq[1] records the case of a boy, aged 13, who, when bathing in a ditch, cut his knee on some broken glass. Tetanus developed seventeen days after. Injections of tetanus antitoxin were commenced on the second day of the disease. Three injections of 10 c.cm. were given—two on successive days, the third at two days' interval after the second. Six days after the commencement of the treatment the spasms had disappeared, but some rigidity remained. This, however, gradually grew less, and the boy returned to perfect health. Other cases of recovery from tetanus in children under antitoxin injections are recorded by Tracey[2] and by Grayson:[3] one in a girl aged 7 years, the other in a boy aged 6½. Both, like that just referred to, were chronic cases. In the girl the disease commenced fourteen days after two slight burns; in the boy, seven days after treading on a nail. Injections of tetanus antitoxic serum were not commenced until seven days after the onset of the disease in the one case, twelve days in the other. Tracey gave twelve injections on an average once a day, Grayson gave five only. Recovery was somewhat slow, but complete in both instances. Withington[4] also records a case of tetanus occurring in a woman, aged 30, after abortion, who recovered after having been subjected to treatment by injections of tetanus antitoxin. The spasms commenced 13 days after the abortion, and the injections were commenced 13 days after the onset of the disease. She was given four doses of 22 c.cm. of the antitoxic serum. Withington discusses the question whether the recovery was spontaneous, or due to the treatment. He quotes figures from Richter and Behring showing the percentage of fatality in cases of tetanus occurring at various periods after injury. Where the incubation-period was under ten days, recoveries in ninety-one cases were only 4 per cent. When the incubation-period extended to between eleven and fifteen days, the number of recoveries was greater. In fifty-four cases it was 27 per cent. When tetanus developed from fifteen to twenty days after injury, in twenty cases the percentage of recoveries was as high as 45 per cent.

1 *Nord méd.*, 1896, iii. 3. 2 *Lancet*, 1896, i. 287.
3 *Pittsburgh M. Rev.*, 1895, ix. 303. Abstract in *Pediatrics*, 1896, i. 92.
4 *Boston M. & S. J.*, 1896, cxxxiv. 53.

Withington mentions that in this case the incubation-period was thirteen days, and since the probability of recovery was one in four, he felt unable to say that the cure was due to the treatment. The same reasoning may be applied to the cases of Tracey and Delbecq, in which the incubation-periods were fourteen days and seventeen days respectively. The only case that comes under the category of those in which the mortality is so high that recovery may be considered to be almost hopeless is that of Grayson, where the tetanus commenced eight days after the injury. It must be remarked, however, that the symptoms were not severe. It is obvious that before we are able to judge the value of tetanus antitoxin, it will be necessary to know how many cases are treated unsuccessfully with this remedy. Since recovery may occur in chronic tetanus under various systems of treatment, the occasional record of probable cure by serum injections will not materially advance the subject.

Hektoen[1] records a case of **interstitial myocarditis due to syphilis** in a child six weeks old, and mentions that only eleven other cases have been recorded. In two of these eleven cases sudden death occurred when the children were considered to be in good health—a noteworthy fact, since it shows that this disease in the child may lead to the same abrupt arrest of heart-action that frequently occurs in the adult when the heart is affected with syphilis. Jacquinet[2] treats the subject of **syphilis of the heart** very fully. In connection with the above remark it may be mentioned that he quotes Mracek as saying that of fifty-eight cases of syphilis of the heart, twenty-one ended in sudden death. Others terminated in what French writers call acute asystole, where severe dyspnœa ushers in the rapidly-approaching end. Jacquinet quotes as an example the case of a prostitute who was dining in a beerhouse with some of her companions, when she complained of pain in the stomach and abdomen. The pains increased, and palpitation of the heart was added. She was removed to a hospital, and died of "advanced asphyxia" after a few hours. The pain mentioned in this case suggests angina pectoris, which may sometimes be epigastric in situation. Jacquinet comments upon this point, and refers to the possibility of cardiac pain being a symptom of syphilis of the heart. He mentions that one of the recorded cases of sudden death occurred in a sailor, who died putting his hand to his heart as if he suffered pain in that region. Huchard is quoted as saying that of 110 cases of angina pectoris, in thirty-two a history of syphilis was obtained, and other observers are mentioned as having noticed severe cardiac pain in syphilitic subjects. This point is of some interest, since potassium iodide is recognised as of value in angina pectoris. The drug is not generally given,

[1] *J. Path. & Bacteriol.*, 1896, iii. 472. [2] *Gaz. d. hôp.*, 1895, lxviii. 917.

however, with the idea of combating syphilis, but of influencing the diseased condition of the coronary arteries that often exists. Yet a satisfactory result naturally suggests that this disease of the coronary arteries may be sometimes syphilitic, like aortitis of the intra-pericardial portion of the aorta, with which cardiac pain is also often associated. Loomis[1] also writes upon the subject of syphilitic lesions of the heart. Fifteen cases of fibroid disease of the heart have come under his observation, three of which were considered beyond all doubt to have been of syphilitic origin. He also has seen four cases of gummata of the heart-wall. Sudden death occurred in two of these cases. Notes are given of one. An apparently healthy man, aged 35, was found lying dead on his bedroom floor, with his hat in his hand, having obviously fallen immediately after entry. The two cases that did not terminate suddenly were in young prostitutes. One of these died with intense dyspnœa and cyanosis; the other was admitted to the Bellevue Hospital for lobar pneumonia, which ended fatally. Dr. Loomis emphasises the point that the question of syphilis as a probable cause of heart-disease should not be overlooked. He says: "When symptoms of cardiac failure occur during the prime of life for which no cause can be ascertained, such as rheumatic history, valvular disease, arterial changes, or kidney complications, especially in one with a syphilitic history, these should always suggest syphilis as the cause of the condition." Before leaving this subject, we may add that Dr. Shingleton Smith[2] has recently recorded a case of enlarged heart, with an extensive fibroid patch in the left ventricle, apparently produced by a gumma, since there was a gumma on the liver and extensive scarring the result of the presence of others. This case, which occurred in a woman aged 38, who apparently had led the life of a prostitute, is interesting, because she had suffered from symptoms pointing to the heart for nearly two years, and had been admitted to the Bristol Royal Infirmary on four separate occasions for cardiac disease during that time. As we have seen, sudden death is common when syphilis attacks the heart; but the more gradual cardiac failure present in such a case as this shows that the disease may sometimes give warning of its presence in sufficient time to render it possible that it may be combated successfully, if we do not habitually ignore its power to injure this important organ.

Dr. Handfield-Jones[3] has delivered the Harveian lectures, taking for his subject **the heart in relation to pregnancy, parturition, and the puerperal state.** It is not possible here to follow carefully all the various points brought out, but one or two of interest may be referred to. The dangers of a defective circulation may be ranged under two heads—dangers primarily to the fœtus and to the mother. In the first place, any derange-

1 *Am. J. M. Sc.*, 1895, cx. 389. 2 *Clin. J.*, 1896, vii. 145.

3 *Lancet*, 1896, i. 145, 213, 275.

ment of the circulation that affects the nutrition of the tissues of the mother affects the placenta, and abortion may take place; on the other hand, the increased work entailed on nourishing the fœtus puts additional strain upon the heart of the mother, which, if weak, may show signs of failure. In the healthy pregnant woman the heart meets the extra requirements by some degree of hypertrophy, which, however, largely disappears as soon as the pregnancy is over and the need for it is removed. If this hypertrophy fails to take place, the heart, which may be sufficiently strong under ordinary circumstances, gives way and cardiac distress makes its appearance. Where heart-weakness has led to repeated abortions, Dr. Handfield-Jones thinks that ordinary care in husbanding the energies of the mother may prevent this accident, and he gives notes of cases illustrating the success that may attend this care. As already mentioned, the danger may, however, be not to the fœtus but to the mother. In rare instances sudden death occurs during parturition, and in others slower cardiac failure occurs a few days after the labour is over. Notes of several cases that have come under his observation are given. In only two is there mention of the result of a *post-mortem* examination; but in both of these valvular disease was absent. Before appreciating the conclusions at which Dr. Handfield-Jones arrives, on reading the brief record of the cases given where symptoms of heart-weakness complicated pregnancy, one may perhaps be struck with the uncertainty of the existence of valvular disease in many of them. Dr. Handfield-Jones lays stress upon this point. His paper seems to be partly intended to show that the hypertrophy of the heart which normally occurs during pregnancy may occasionally be defective. A nutrition that ought to be physiological may be so altered as to be pathological, and cardiac failure of various degrees occurs even when the valves are perfectly healthy. Of the various forms of valvular disease, mitral stenosis is considered the most serious; but Dr. Handfield-Jones does not think that it necessarily adds to the risks of child-bearing. One woman with an obviously diseased heart may have several children without serious consequences; another, whose heart appears healthy before the commencement of pregnancy, may die of cardiac failure after labour.

Other papers have recently appeared upon heart-disease in pregnancy. Allyn [1] of Philadelphia gives a *resumé* of published literature upon this subject, and adopts the view that mitral stenosis is a serious lesion, but thinks that if well compensated, and there is no subjective evidence of its presence, marriage may be permitted. Rosenberg, [2] on the other hand, who records two cases of death in primiparæ affected with heart-disease, states that the presence of such disease makes it imperative that marriage should be forbidden.

THEODORE FISHER.

[1] *Univ. M. Mag.*, 1896, viii. 172. [2] *N. York M. J.*, 1896, lxiii. 77.

